# Drug resistance characteristics of *Mycobacterium tuberculosis* isolates between 2014 and 2017 in Sichuan, China: A retrospective study

**DOI:** 10.1371/journal.pone.0209902

**Published:** 2018-12-31

**Authors:** Mi Zhou, Shan Liu, Qingfeng Li, Qiming Wang, Ma Zhu, Ling Cao, Dongmei Wang, Yuanhong Xu, Tianli Zheng, Qian Ye, Xiuying Hu, Haojiang Zuo, Xiaofang Pei

**Affiliations:** 1 Department of Public Health Laboratory Sciences, West China School of Public Health, Sichuan University, Chengdu, Sichuan Province, China; 2 Department of Clinical Laboratory, Public Health Clinical Center of Chengdu, Chengdu, Sichuan Province, China; 3 Department of Clinical Laboratory, Affiliated Hospital of University of Electronic Science and Technology, Sichuan Academy of Medical Sciences and Sichuan Provincial People’s Hospital, Chengdu, Sichuan Province, China; 4 No.4 West China Teaching Hospital, Sichuan University, Chengdu, Sichuan Province, China; 5 West China Hospital/West China School of Nursing, Sichuan University, Chengdu, Sichuan Province, China; Jamia Hamdard, INDIA

## Abstract

**Background:**

The prevalence of drug-resistant tuberculosis (DR-TB) has brought severe challenges to the prevention and control of tuberculosis. Studies have explored the status of antituberculosis drug (ATD) resistance in different regions of China. However, few studies have focused on DR-TB in Sichuan to date. Due to the large population in Sichuan, detailed investigations of the DR-TB burden in Sichuan are needed. The objective of this study was to investigate the drug resistance characteristics of TB isolates from tuberculosis patients with and without HIV (TB-HIV patients and TBw/oHIV patients) in Chengdu, Sichuan, China.

**Methods:**

Isolates from respiratory samples of TBw/oHIV patients and TB-HIV patients hospitalized between January 2014 and December 2017 were collected. Nontuberculosis mycobacteria (NTM) were excluded. Drug sensitivity testing (DST) was performed according to the dilution method in microplates with 4 first-line ATDs and 8 second-line ATDs. TB strains were separated according to patient treatment history, patient age, calendar year and GeneXpert MTB/RIF (GeneXpert) assay results for further analysis.

**Results:**

For the 7470 patients recruited, the multidrug-resistant tuberculosis (MDR-TB) rate was 2.1-fold (14.6% vs. 6.8%) higher than the national baseline level. The repeatedly admitted patients were more likely to have a resistance profile than the first-time-admitted cases in both the TB-only group (*P*<0.05) and the TB-HIV corresponding group (*P*<0.05). Among the 7273 TBw/oHIV cases and 197 TB-HIV cases, the positivity rates of acid-fast bacilli (AFB) in the TB-HIV group were significantly lower than those in the TBw/oHIV group (*P*<0.05). The repeatedly admitted TB-HIV patients had lower resistance rates to INH than the repeatedly admitted TBw/oHIV patients (24.4% vs. 41.5%, *P*<0.05). The Rifampicin-resistant TB strains in the TBw/oHIV group were more likely to be resistant to INH in the repeatedly admitted group than those in the first-time admitted patients (*P*<0.05). The proportions of XDR (3.6% vs. 1.3%, *P*<0.05) and XDR-TB/MDR-TB (7.3% vs. 2.2%, P<0.05) in all TB-HIV patients were significantly higher than those in all TBw/oHIV patients. The ratio of XDR-TB was significantly higher in the TB-HIV group than in the TBw/oHIV group (30.4% vs. 9.0%, *P*<0.05) and the all TB group (9.0% vs. 10.1%, *P*<0.05). Regarding age, the <25-year-old TB-HIV patients (9.1% vs. 0.7%, *P*<0.05) and 25~44-year-old TB-HIV patients (5.2% vs. 2.4%, *P*<0.05) were more likely to have a higher XDR proportion than their TBw/oHIV counterparts. The ATD-resistance profile in terms of different years from high to low was 2014>2015>2016≈2017 for TBw/oHIV patients. The same trend was also observed for TB-HIV patients: 2014>2015>2016≈2017. The GeneXpert TB-positive rate in the TBw/oHIV group was higher than that in the TB-HIV group [81%(639/792) vs. 65% (13/20), *P*<0.05]. In TBw/oHIV cases, the agreement was 92.3% and the Kappa value was 0.75. In TB-HIV cases, the agreement was 85.0% and the Kappa value was 0.32.

**Conclusion:**

In Sichuan, ATD resistance has improved since 2014, but to date, it remains severe. The different resistance profiles of TBw/oHIV patients and TB-HIV patients indicates the need for personalized treatment plans. Specifically, the GeneXpert assay might be more suitable for TBw/oHIV patients than for TB-HIV patients.

## Introduction

Drug-resistant tuberculosis (DR-TB), especially multidrug-resistant tuberculosis (MDR-TB), has become a serious public health problem for the effective control of tuberculosis. China is one of several countries worldwide with a heavy burden of MDR-TB patients [1]. Extensive efforts have been made to prevent and control MDR-TB. Although China has achieved 100% coverage for the Directly Observed Treatment Short course (DOTS) strategy since 2007, the DOTS execution quality and DR-TB epidemic status have varied. Recently, the nationwide first-line antituberculosis drug (ATD) resistance rate was 36.8% [2]. While this number varied in different regions, in northeast China, it was 30.4% [3]; in Xinjiang, it was 22.7% [4]; and in Lhasa, Tibet, it was 63.1% [5]. Thus, local drug resistance surveillance is important to evaluate the tuberculosis epidemic status in different regions.

Sichuan is located in southwest China, is a mountainous area and comprises a large migrant population. A recent local study indicated that the rate of MDR-TB patients in Sichuan was higher than the national level and higher than that in the fifth epidemiological investigation in Sichuan during 2010 [6]. However, surveillance data on the proportion of MDR-TB patients in Sichuan is limited [7]. Moreover, TB is one of the most common presenting illnesses and the leading cause of death among people living with HIV [8]. Until now, both TB-HIV coinfected patients (TB-HIV patients) and TB patients without HIV (TBw/oHIV patients) have been administered the same ATDs. TB-HIV patients have a higher risk of drug-related side effects and toxicity, which requires close monitoring and special care. The interactions between antiviral drugs and ATDs need more attention. To date, little is known about DR-TB isolated from HIV patients. The DR-TB and XDR-TB data in Sichuan need to be updated. Therefore, the aim of this study was to present the current drug resistance profiles of M. tuberculosis in Sichuan province by means of a retrospective analysis from January 2014 to December 2017.

## Materials and methods

### Ethics statement

This research was approved by the Ethics Committee of Public Health Clinical Center of Chengdu (No.2014Y-14 and No.2014Y-21). Because patient records were anonymized prior to the analysis, no consent was obtained.

### Study design

The study was a retrospective study analyzing routinely collected laboratory data.

### Study population

Sichuan province lies in southwest China and is one of China’s largest provinces, with a population of 89 million. This study was carried out at the Public Health Clinical Center of Chengdu (PHCCC). This institution is the authorized hospital for mainly treating TB and HIV patients, and it is the leading hospital of the Tuberculosis Hospitals Union in southwest China.

To avoid selection bias, this retrospective study enrolled consecutive culture-positive Mycobacterium tuberculosis cases that were confirmed and treated in the PHCCC between January 2014 and December 2017. HIV was diagnosed based on Chinese HIV and HIV Infection Diagnostic Criteria (WS293-2008). TB was diagnosed based on the Chinese Pulmonary Tuberculosis Diagnostic Criteria (WS 288–2017), the Chinese ‘*TB volume of clinical diagnosis and treatment guidelines*’ (Chinese Medical Association, 2005) and the updated WHO guidelines [9]. A total of 8087 strains with DST and species identification were collected in this study. Nontuberculosis mycobacteria (NTM) were excluded from this study. TB strains were separated by patient treatment history, patient age, and calendar year for further analysis.

### *M*. *tuberculosis* culture

Respiratory samples, including sputum and bronchoscopy fluid, were collected from patients with suspected TB with or without HIV for mycobacterial testing by mycobacterial culture with the BD BACTEC MGIT 960 Culture System (Becton Dickinson & Co., Franklin Lakes, NJ, USA).

### Sample preparation

Sample preparation was carried out in a biological safety cabinet. Samples, including sputum and bronchoscopy fluid, were transferred to 50-mL tubes. The volume of the specimen was maintained at less than 10 mL; the same amount of 2% NALC-NaOH treatment solution was added. The sample was placed on a vortex shaker for 1 min to facilitate sample digestion. Then, the tube was kept in a 37°C oscillating water bath for approximately 3 min. Sterile PBS (pH 6.8) was added to a total volume of 50 mL. Then, the tube was centrifuged at 3000 g for 15 min. The supernatant was discarded. Approximately 2 mL of PBS (pH 6.8) was added to resuspend the pellet. The sample was used for the MGIT 960 liquid culture inoculation, for the Roche solid culture inoculation, and for smear staining.

### Drug sensitivity testing (DST)

The main drug sensitivity testing (DST) was conducted as follows: To avoid biosafety hazards, the DST was performed in a biological safety cabinet. The DST kit was obtained from AUTOBIO diagnostics Co., Ltd. (Zhengzhou, China). According to the standard procedure of the manufacturer, each TB bacterial sample was first diluted to 1 McFarland unit (1 McFarland = 1 mg/mL) and then diluted with culture medium to the working concentration (10^−2^ mg/mL).

In a 36-well plate, one well (usually the C7 well) was set as the p-nitrobenzoic acid (PNB) well. Another well (usually the C8 well) was set as the thiophene-2-carboxylic acid hydrazide (TCH) well. Another two wells (usually the D7 well and D8 well) were set as the reference (Ref) wells, which contained 500 μL of bacterial solution (10^−2^ mg/mL). The remaining wells contained different drugs. Every two wells were for one drug; one well had a low concentration, and the other had a medium concentration. The drugs were used as follows: isoniazid (INH, 0.12 μg/mL and 0.25 μg/mL), rifampicin (RIF, 1.0 μg/mL and 2.0 μg/mL), streptomycin (STR, 1.0 μg/mL and 2.0 μg/mL) and ethambutol (EMB, 2.5 μg/mL and 5.0 μg/mL), and the 8 second-line drugs, including the fluoroquinolone drugs (Ofloxacin (OFX, 1.5 μg/mL and 2.0 μg/mL), levofloxacin (LFX, 1.5 μg/mL and 2.0 μg/mL), and moxifloxacin (MFX, 0.5 μg/mL and 1.0 μg/mL); the oral bacteriostatic second-line ATDs (Prothionamide/Ethionamide (PTO/ETO, 2.5 μg/mL and 5.0 μg/mL) and Rifabutin (RFB, 0.5 μg/mL and 0.5 μg/mL); the second-line parenteral agents (injectable ATDs) Amikacin (AMK, 1.3 μg/mL and 2.5 μg/mL) and Capreomycin (CM, 1.3 μg/mL and 2.5 μg/mL); and the group 5 drug Clarithromycin (CLR, 2.5 μg/mL and 5.0 μg/mL).

A total of 0.5 mL of bacterial solution (10^−2^ mg/mL) was added into each well. After incubation at 37°C for 5 days, the Ref wells were observed. If 1/3~4/5 of the Ref wells were covered by bacteria, 12 μL of reagent A and 25 μL of reagent B were added into each well for visualization. Then, the wells were observed after incubation at 37°C for approximately 24 h. If the Ref wells were stained red and the PNB well was stained blue, the results were reliable. For the other wells exposed to different drugs, if both the medium-concentration and low-concentration wells were stained blue, the results were reliable. If the medium concentration was stained blue and the low concentration was stained red, the results were reliable. If the medium concentration was stained red and the low concentration was stained red, the result was invalid. For strain identification, both PNB and TCH were blue, indicating that the strain was bovine TB. Both PNB and TCH were red, indicating that the strain was nontuberculosis mycobacteria (NTM). PNB was blue, and TCH was red, indicating that the strain was human TB. Quality control was assessed with interlaboratory confirmation tests and evaluated annually by the national reference laboratory of China. Each batch of new drugs was examined with the reference strain H37Rv (ATCC 27294), which was susceptible to all standard ATDs [10]. During January 2014 to December 2017, all the DST kits used in this study were commercially available kits. In some batches, the concentrations of a few ATDs might have tiny variations. With quality assurance by both the company and the PHCCC, these variations were considered to be acceptable.

### Definitions of drug resistance

The following definitions were used. Drug resistance was defined as resistance to at least one of the aforementioned ATDs. MDR was defined as TB resistance to at least isoniazid and rifampin.

Pre-XDR was defined as resistance to isoniazid, rifampin and either the fluoroquinolones or one of the second-line anti-TB injectable drugs, but not both. XDR was defined as resistance to at least rifampin and isoniazid, as well as to any member of the quinolone family and at least one second-line anti-TB injectable drug (kanamycin, capreomycin, or amikacin).

### GeneXpert MTB/RIF assay

In this study, approximately one-tenth of our patients who were willing to pay approximately 800 RMB for rapid detection received GeneXpert assay. One milliliter of respiratory specimens was mixed with approximately 2 mL of Xpert “Sample Reagent,” shaken for 1 min and digested for 15 min at room temperature with intermittent shaking. After processing, a 2 mL sample was transferred into the open port of the Xpert cartridge. After the test was initiated, reports were automatically generated approximately 1.5 h later.

### Statistical analysis

Data were analyzed using SPSS Statistics Client 19.0 (SPSS Inc., Chicago, IL, USA). Continuous variables were expressed as the median or mean ± standard deviation (S.D.), and categorical variables were expressed as the number and percentage. Comparisons between groups or subgroups were performed using Student's *t*-test for continuous variables, the χ2 test or Fisher's exact test for categorical variables and the Wilcoxon rank-sum test for nonparametric data. The level of significance was set at *P* < 0.05.

## Results

### General information

**Fig 1:**
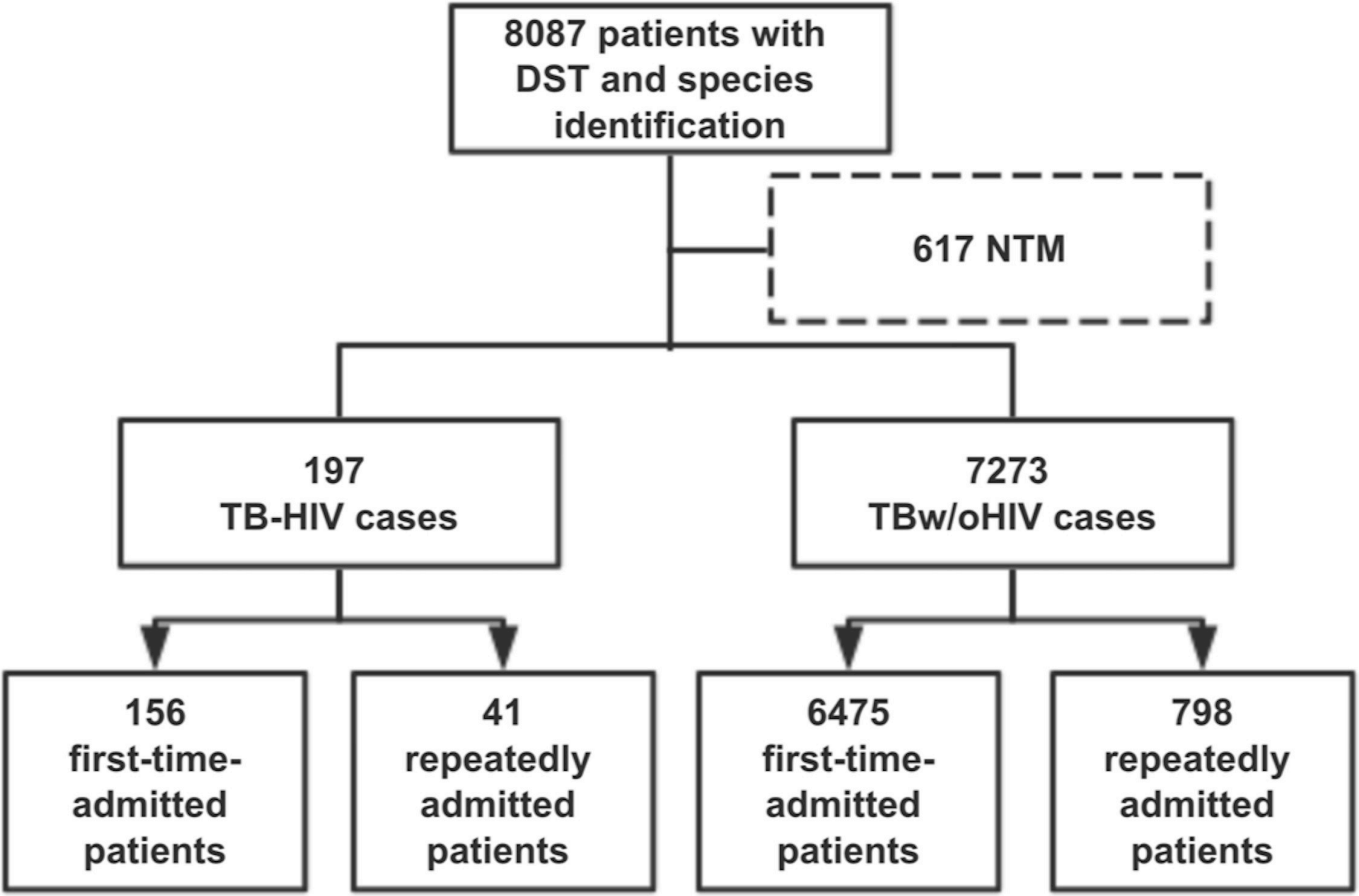
Flow diagram of participant enrollment. DST, drug sensitivity testing; NTM, nontuberculous mycobacteria.

Between January 2014 and December 2017, apart from 617 non-*Mycobacterium tuberculosis* cases, a total of 7470 culture-confirmed *Mycobacterium tuberculosis* cases were reported, of which 197 had HIV coinfections (TB-HIV group) that were clinically diagnosed (**Fig 1**).

There were 4955 men and 2318 women in the TB without HIV infection group (TBw/oHIV group) and 175 men and 22 women in the TB-HIV group. Compared with the TBw/oHIV group, TB-HIV coinfection was higher in men than in women (*P* < 0.05). The mean ± S.D. age of all patients was 42.3±19.2 years. Approximately 88.8% of cases (6631/7470) visited PHCCC for the first time for TB treatment (the first-time-admitted group), and whether these patients received TB treatment previously was not recorded. Approximately 11.2% (839/7470) were repeatedly admitted to PHCCC for TB treatment (the repeatedly admitted group).

### Positivity rates of acid-fast bacilli smears

**Fig 2:**
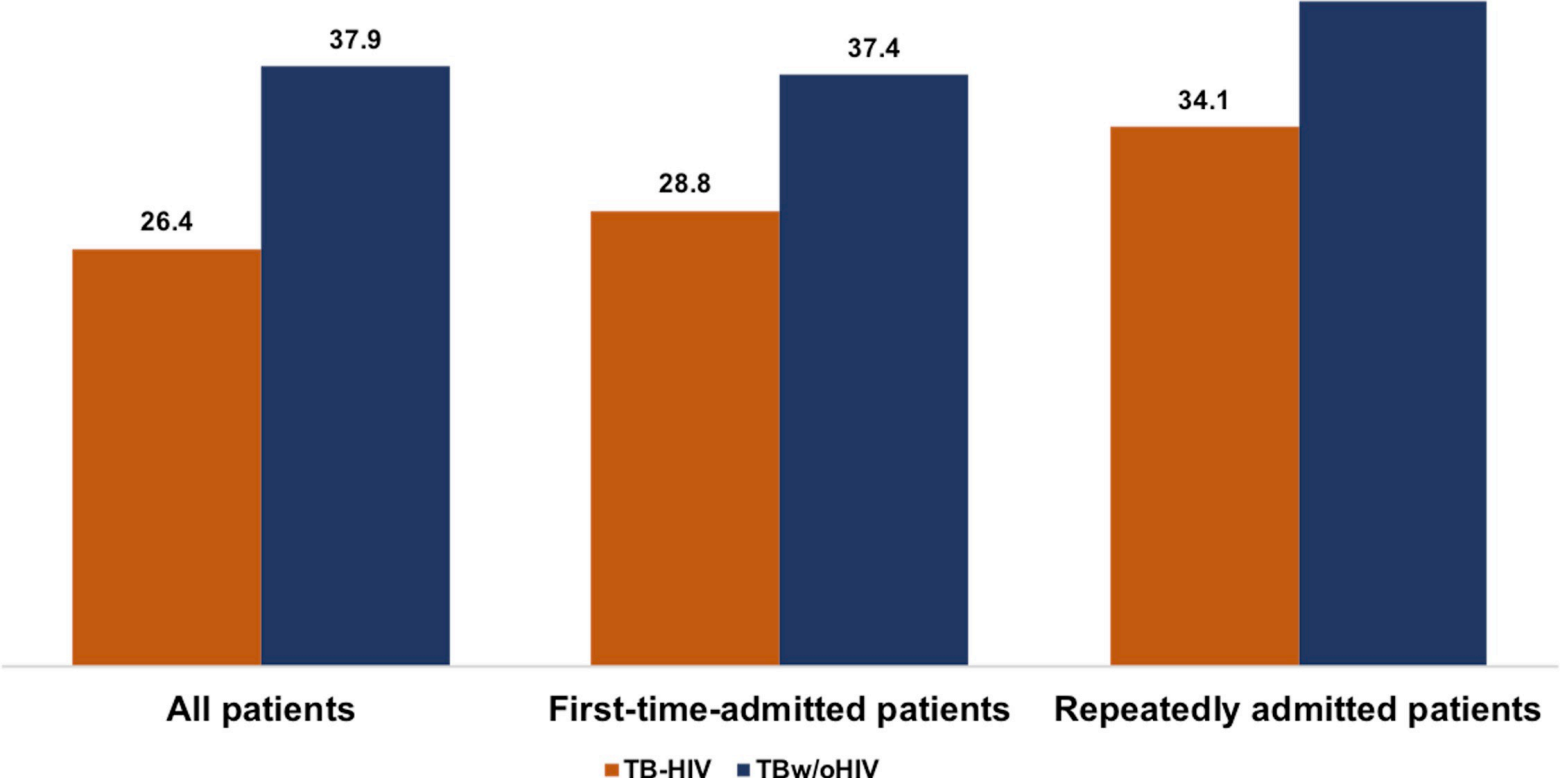
AFB smear positivity rates for the TB-HIV group and TBw/oHIV group.

Significantly lower positivity rates of acid-fast bacilli (AFB) smears were found in all TB-HIV patients (26.4% vs. 37.9%, **Fig 2**), the first-time-admitted TB-HIV patients (28.8% vs. 37.4%, *P*<0.05, **Fig 2**), and the repeatedly admitted TB-HIV patients (17.1% vs. 42.0%, *P*<0.05, **Fig 2**) than in their counterparts in the TBw/oHIV group.

### Overall drug resistance patterns

The drug resistance patterns of the TB-HIV group and TBw/oHIV group are shown separately by treatment history (first-time-admitted patients, repeatedly admitted patients, and all patients) in Table 1.

**Table 1:** Drug resistance patterns of *M*. *tuberculosis* in the TB-HIV coinfection group and the TB without HIV infection group.

Drug resistance	TB-HIV coinfection [*N* (%, 95% CI)]			TB without HIV infection [*N* (%, 95% CI)]		
	First-time-admitted patients ^1^	Repeatedly admitted patients ^2^	All patients ^3^	First-time-admitted patients ^4^	Repeatedly admitted patients ^5^	All patients ^6^
**AFB smear-positive**	45(28.8,21.7~36.0) *	7(17.1,5.4~28.7) *	52(26.4,20.2~32.6) *	2424(37.4,36.3~38.6)	335(42.0,38.6~45.4)	2759(37.9,36.8~39.1)
**Any drug resistance ^#^**	73(46.8,38.9~54.7)	14(34.1,19.5~48.8) *	87(44.2,37.2~51.1) *	3384(52.3,51.0~53.5)	462(57.9,54.5~61.3)	3846(52.9,51.7~54.0)
**Any first-line drug resistance**	35(22.4,15.9~29.0)	10(24.4,11.1~37.7) *	45(22.8,17.0~28.7)	1737(26.8,25.7~27.9)	363(45.5,42.0~48.9)	2100(28.9,27.8~29.9)
**Any second-line drug resistance**	68(43.6,35.8~51.4)	12(29.3,15.2~43.4) *	80(40.6,33.7~47.5)	2829(43.7,42.5~44.9)	376(47.1,43.7~50.6)	3205(44.1,42.9~45.2)
**INH**	29(18.6,12.5~24.7)	10(24.4,11.1~37.7) *	39(19.8,14.2~25.4)	1383(21.4,20.4~22.4)	331(41.5,38.1~44.9)	1714(23.6,22.6~24.5)
**RIF**	20(12.8,7.6~18.1)	8(19.5,7.2~31.8)	28(14.2,9.3~19.1)	1003(15.5,14.6~16.4)	255(32.0,28.7~35.2)	1258(17.3,16.4~18.2)
**STR**	13(8.3,4.0~12.7)	6(14.6,3.7~25.6)	19(9.6,5.5~13.8)	838(12.9,12.1~13.8)	189(23.7,20.7~26.6)	1027(14.1,13.3~14.9)
**EMB**	1(0.6,-0.6~1.9)	1(2.4,-2.3~7.2)	2(1.0,-0.4~2.4)	97(1.5,1.2~1.8)	24(3.0,1.8~4.2)	121(1.7,1.4~2.0)
**OFX**	12(7.7,3.5~11.9)	7(17.1,5.4~28.7)	19(9.6,5.5~13.8)	569(8.8,8.1~9.5)	158(19.8,17.0~22.6)	727(10.0,9.3~10.7)
**LFX**	10(6.4,2.6~10.3)	6(14.6,3.7~25.6)	16(8.1,4.3~11.9)	447(6.9,6.3~7.5)	123(15.4,12.9~17.9)	570(7.8,7.2~8.5)
**AMK**	4(2.6,0.1~5.1)	2(4.9,-1.8~11.6)	6(3.0,0.6~5.5)	81(1.3,1.0~1.5)	41(5.1,3.6~6.7)	122(1.7,1.4~2.0)
**CM**	2(1.3,-0.5~3.1)	3(7.3,-0.8~15.4) *	5(2.5,0.3~4.7) *	49(0.8,0.5~1.0)	14(1.8,0.8~2.7)	63(0.9,0.7~1.1)
**PTO/ETO**	52(33.3,25.9~40.8)	6(14.6,3.7~25.6)	58(29.4,23.1~35.8)	2202(34.0,32.9~35.2)	206(25.8,22.8~28.9)	2408(33.1,32.0~34.2)
**MFX**	15(9.6,5.0~14.3)	6(14.6,3.7~25.6)	21(10.7,6.3~15.0)	548(8.5,7.8~9.1)	161(20.2,17.4~23.0)	709(9.7,9.1~10.4)
**RFB**	14(9.0,4.5~13.5)	7(17.1,5.4~28.7)	21(10.7,6.3~15.0)	696(10.7,10.0~11.5)	194(24.3,21.3~27.3)	890(12.2,11.5~13.0)
**CLR**	1(0.6,-0.6~1.9)	0(0.0,-)	1(0.5,-0.5~1.5)	50(0.8,0.6~1.0)	25(3.1,1.9~4.3)	75(1.0,0.8~1.3)
**pre-XDR**	4(2.6,0.1~5.1)	3(7.3,-0.8~15.4)	7(3.6,1.0~6.1)	294(4.5,4.0~5.0)	101(12.7,10.3~15.0)	395(5.4,4.9~6.0)
**XDR**	4(2.6,0.1~5.1) *	3(7.3,-0.8~15.4)	7(3.6,1.0~6.1) *	65(1.0,0.8~1.2)	31(3.9,2.5~5.2)	96(1.3,1.1~1.6)
**MDR (INH+RIF)**	15(9.6,5.0~14.3)	8(19.5,7.2~31.8)	23(11.7,7.2~16.2)	832(12.8,12.0~13.7)	233(29.2,26.0~32.4)	1065(14.6,13.8~15.5)
**INH+STR**	11(7.1,3.0~11.1)	6(14.6,3.7~25.6)	17(8.6,4.7~12.6)	639(9.9,9.1~10.6)	178(22.3,19.4~25.2)	817(11.2,10.5~12.0)
**INH+RIF+STR**	9(5.8,2.1~9.4)	5(12.2,2.1~22.3)	14(7.1,3.5~10.7)	473(7.3,6.7~7.9)	149(18.7,16.0~21.4)	622(8.6,7.9~9.2)
**INH+RIF+EMB**	1(0.6,-0.6~1.9)	1(2.4,-2.3~7.2)	2(1.0,-0.4~2.4)	93(1.4,1.1~1.7)	22(2.8,1.6~3.9)	115(1.6,1.3~1.9)
**RIF+STR+EMB**	1(0.6,-0.6~1.9)	1(2.4,-2.3~7.2)	2(1.0,-0.4~2.4)	71(1.1,0.8~1.4)	20(2.5,1.4~3.6)	91(1.3,1.0~1.5)
**INH+RIF+STR+EMB**	9(5.8,2.1~9.4)	5(12.2,2.1~22.3)	14(7.1,3.5~10.7)	497(7.7,7.0~8.3)	152(19.0,16.3~21.8)	649(8.9,8.3~9.6)

TB, tuberculosis; HIV, human immunodeficiency virus; DST, drug sensitivity testing; NH, isoniazid; STR, streptomycin; RIF, rifampicin; EMB, ethambutol; OFX, Ofloxacin; LFX, Levofloxacin; MFX, Moxifloxacin; PTO/ETO, Protionamide/Ethionamide; RFB, Rifabutin; AMK, Amikacin; CM, Capreomycin; CLR, Clarithromycin; MDR-TB, multidrug-resistant tuberculosis.

^1^ TB-HIV coinfected patients, the first visit to PHCCC for treatment, *n* = 156;

^2^ TB-HIV coinfected patients repeatedly admitted to PHCCC for treatment, *n* = 41;

^3^ All TB-HIV coinfected patients who attended PHCCC for treatment, *n* = 197;

^4^ TBw/oHIV patients, the first visit to PHCCC for treatment, *n* = 6475;

^5^ TBw/oHIV patients repeatedly admitted to PHCCC for treatment, *n* = 798;

^6^ All TBw/oHIV patients who attended PHCCC for treatment, *n* = 7273.

^#^: Resistant to at least one drug.

*: *P*<0.05 vs. TBw/oHIV group.

Regarding the first-line ATDs, the repeatedly admitted TB-HIV patients had lower resistance rates to INH than the repeatedly admitted TBw/oHIV patients (24.4% vs. 41.5%, *P*<0.05, Table 1). Moreover, the RIF-resistant TB strains in the TBw/oHIV group were more likely to also be resistant to INH in the repeatedly admitted group than in the first-time admitted group (*P*<0.05, Table 1).

Regarding the second-line ATDs, the drug resistance rates to CM in all TB-HIV patients (2.5% vs. 0.9% *P*<0.05) and the repeatedly admitted TB-HIV patients (7.3% vs. 1.8%, *P*<0.05) were significantly higher than those in the TBw/oHIV group.

Moreover, a total of 103 cases were confirmed to be XDR-TB (1.4% of 7470 culture-confirmed TB cases; 9.5% of 1088 MDR-TB cases, Table 1).

The proportion of XDR in all TB-HIV patients was statistically higher than that in all TBw/oHIV patients (3.6% vs. 1.3%, *P*<0.05). Subgroup analysis showed that the first-time-admitted TB-HIV patients also had a higher XDR rate (2.6% vs. 1.0%, *P*<0.05) than their TBw/oHIV counterparts. The results also demonstrated that the proportion of MDR-TB cases with XDR-TB (XDR-TB/MDR-TB) among all TB-HIV patients was significantly higher than among the TBw/oHIV patients (7.3% (7/96) vs. 2.2% (23/1065), *P*<0.05). Subgroup analysis showed that the first-time-admitted TB-HIV patients also had a higher proportion of XDR/MDR (6.2% (4/65) vs. 1.8% (15/832), *P*<0.05) than their TBw/oHIV counterparts.

**Fig 3:**
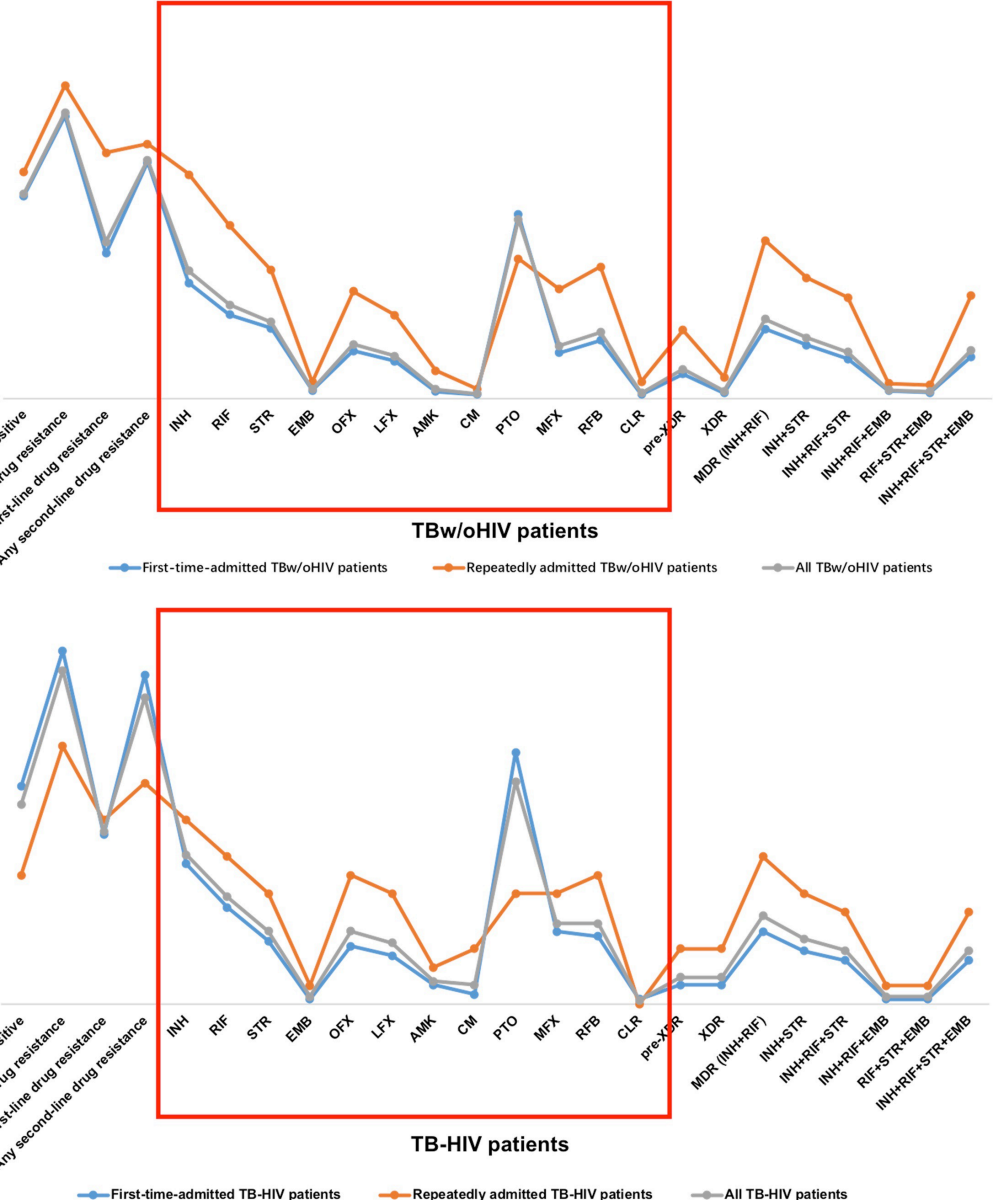
ATD profiles of patients with different treatment histories. **3A**: ATD profiles of TB-HIV groups with different treatment histories. **3B**: ATD profiles of TBw/oHIV groups with different treatment histories.

When compared to the whole profile containing 4 first-line ATDs and 8 second-line ATDs, the Wilcoxon rank-sum test revealed that the repeatedly admitted patients were more likely to have a resistance profile than the first-time-admitted cases in both the TBw/oHIV group (*P*<0.05, **Fig 3A**) and the corresponding TB-HIV group (*P*<0.05, **Fig 3B**). No significant difference was found between the first-time-admitted TB-HIV patients and the first-time-admitted TBw/oHIV patients. However, significantly lower ATD resistance was observed in the repeatedly admitted TB-HIV patients than in the repeatedly admitted TBw/oHIV patients using the Wilcoxon rank-sum test (*P*<0.05, Table 1).

### Drug resistance profiles of different age groups

Patients of different ages were mainly divided into 4 groups: the <25-year-old group, the 25~44-year-old group, the 45~64-year-old group and the >65-year-old group.

As shown in Table 2, when the TBw/oHIV patients and the TB-HIV patients were compared, the <25-year-old TB-HIV patients had a significantly higher XDR proportion (9.1% vs. 0.7%, *P*<0.05, Table 2) than the <25-year-old TBw/oHIV patients. The 25~44-year-old TB-HIV patients had a significantly higher XDR proportion (5.2% vs. 2.4%, *P*<0.05, Table 2) than the 25~44-year-old TBw/oHIV patients. In contrast, the 45- to 64-year-old TB-HIV patients had a significantly lower proportion of any drug resistance rate (21.3% vs. 44.6%, *P*<0.05, Table 2), any first-line drug resistance (41.0% vs. 55.4%, *P*<0.05, Table 2), RIF (6.6% vs. 18.5%, *P*<0.05, Table 2), STR (3.3% vs. 13.9%, *P*<0.05, Table 2), MDR (INH+RIF) (6.6% vs. 15.8%, *P*<0.05, Table 2), INH+STR (1.6% vs. 11.1%, *P*<0.05, Table 2), and INH+RIF+STR+EMB (1.6% vs. 8.5%, *P*<0.05, Table 2).

**Table 2:** Drug-resistant distribution of Mycobacterium tuberculosis isolates in different age groups.

Drug resistance	TB-HIV coinfection [*N* (%, 95% CI)]				TB without HIV infection [*N* (%, 95% CI)]			
	<25 ^1^	25~44 ^2^	45~64 ^3^	>64 ^4^	<25 ^5^	25~44 ^6^	45~64 ^7^	>64 ^8^
AFB smear-positive	1(9.1,-8.7~26.9)	35(30.4,22~38.9)	13(21.3,10.9~31.7) *	3(30,0.1~59.9)	517(29.3,27.2~31.5) δ	815(36.0,34.0~37.9) α,χ,δ	938(44.6,42.5~46.8) α	489(42.8,39.9~45.7)
Any drug resistance #	3(27.3,-0.3~54.9)	55(47.8,38.7~57)	25(41,28.5~53.4) *	4(40,8~72)	910(51.6,49.3~54.0) δ	1283(56.6,54.6~58.6) α,δ	1163(55.4,53.2~57.5) α,δ	490(42.9,40.0~45.7)
Any first-line drug resistance	2(18.2,-5.7~42.1)	29(25.2,17.2~33.2)	11(18,8.3~27.8) *	3(30,0.1~59.9)	454(25.8,23.7~27.8) δ	758(33.4,31.5~35.4) α,δ	6569(31.2,29.2~33.2) α,δ	232(20.3,18.0~22.6)
Any second-line drug resistance	3(27.3,-0.3~54.9)	49(42.6,33.5~51.7)	24(39.3,27~51.7)	4(40,8~72)	758(43.0,40.7~45.3) δ	1089(48.0,46.0~50.1) α,δ	959(45.6,43.5~47.8)	399(34.9,32.1~37.7)
INH	2(18.2,-5.7~42.1)	25(21.7,14.2~29.3)	10(16.4,7~25.8)	2(20,-6.1~46.1)	335(19.0,17.2~20.8)	639(28.2,26.3~30.0) α,δ	546(26.0,24.1~27.9) α,δ	194(17.0,14.8~19.2)
RIF	1(9.1,-8.7~26.9)	21(18.3,11.2~25.4)	4(6.6,0.3~12.8) *	2(20,-6.1~46.1)	257(14.6,12.9~16.2) δ	495(21.8,20.1~23.5) α,χ,δ	389(18.5,16.9~20.2) α,δ	117(10.2,8.5~12.0)
STR	1(9.1,-8.7~26.9)	14(12.2,6.2~18.2)	2(3.3,-1.2~7.8) *	2(20,-6.1~46.1)	248(14.1,12.5~15.7) δ	401(17.7,16.1~19.3) α,χ,δ	293(13.9,12.5~15.4) δ	85(7.4,5.9~9.0)
EMB	0(0.0,-)	2(1.7,-0.7~4.1)	0(0,-)	0(0,-)	24(1.4,0.8~1.9)	52(2.3,1.7~2.9) α,δ	32(1.5,1.0~2.0)	13(1.1,0.5~1.8)
OFX	1(9.1,-8.7~26.9)	15(13,6.9~19.2)	3(4.9,-0.6~10.4)	0(0,-)	119(6.8,5.6~7.9)	275(12.1,10.8~13.5) α,δ	237(11.3,9.9~12.6) α,δ	96(8.4,6.8~10.0)
LFX	1(9.1,-8.7~26.9)	13(11.3,5.5~17.1)	2(3.3,-1.2~7.8)	0(0,-)	97(5.5,4.4~6.6)	219(9.7,8.4~10.9) α,δ	178(8.5,7.3~9.7) α	76(6.6,5.2~8.1)
AMK	1(9.1,-8.7~26.9)	5(4.3,0.6~8.1)	0(0,-)	0(0,-)	16(0.9,0.5~1.4)	63(2.8,2.1~3.5) α,δ	33(1.6,1.0~2.1)	10(0.9,0.3~1.4)
CM	0(0.0,-)	4(3.5,0.1~6.8)	1(1.6,-1.6~4.9)	0(0,-)	10(0.6,0.2~0.9)	31(1.4,0.9~1.8) α,δ	15(0.7,0.4~1.1)	7(0.6,0.2~1.1)
PTO/ETO	1(9.1,-8.7~26.9)	36(31.3,22.8~39.8)	19(31.1,19.4~42.9)	2(20,-6.1~46.1)	603(34.2,32.0~36.4) δ	798(35.2,33.2~37.2) δ	705(33.6,31.5~35.6) δ	302(26.4,23.9~29.0)
MFX	1(9.1,-8.7~26.9)	16(13.9,7.6~20.3)	4(6.6,0.3~12.8)	0(0,-)	114(6.5,5.3~7.6) δ	263(11.6,10.3~12.9) α,δ	230(10.9,9.6~12.3) α	102(8.9,7.3~10.6)
RFB	2(18.2,-5.7~42.1)	14(12.2,6.2~18.2)	3(4.9,-0.6~10.4)	2(20,-6.1~46.1)	182(10.3,8.9~11.8) δ	375(16.5,15.0~18.1) α,χ,δ	258(12.3,10.9~13.7) δ	75(6.6,5.1~8.0)
CLR	0(0.0,-)	1(0.9,-0.8~2.6)	0(0,-)	0(0,-)	11(0.6,0.3~1.0)	31(1.4,0.9~1.8) α	20(1.0,0.5~1.4)	13(1.1,0.5~1.8)
pre-XDR	0(0.0,-)	5(4.3,0.6~8.1)	2(3.3,-1.2~7.8)	0(0,-)	76(4.3,3.4~5.3)	156(6.9,5.8~7.9) α,δ	126(6.0,5.0~7.0) α,δ	37(3.2,2.2~4.3)
XDR	1(9.1,-8.7~26.9) *	6(5.2,1.1~9.3) *	0(0,-)	0(0,-)	12(0.7,0.3~1.1)	54(2.4,1.8~3.0) α,χ,δ	23(1.1,0.6~1.5)	7(0.6,0.2~1.1)
MDR (INH+RIF)	1(9.1,-8.7~26.9)	17(14.8,8.3~21.3)	4(6.6,0.3~12.8) *	1(10,-9.6~29.6)	209(11.9,10.4~13.4) δ	430(19.0,17.4~20.6) α,χ,δ	332(15.8,14.2~17.4) α,δ	94(8.2,6.6~9.8)
INH+STR	1(9.1,-8.7~26.9)	13(11.3,5.5~17.1)	1(1.6,-1.6~4.9) *	2(20,-6.1~46.1)	172(9.8,8.4~11.1) δ	342(15.1,13.6~16.6) α,χ,δ	233(11.1,9.7~12.4) δ	70(6.1,4.7~7.5)
INH+RIF+STR	1(9.1,-8.7~26.9)	11(9.6,4.2~15)	1(1.6,-1.6~4.9)	1(10,-9.6~29.6)	131(7.4,6.2~8.7) δ	276(12.2,10.8~13.5) α,χ,δ	170(8.1,6.9~9.3) δ	45(3.9,2.8~5.1)
INH+RIF+EMB	0(0.0,-)	2(1.7,-0.7~4.1)	0(0,-)	0(0,-)	22(1.2,0.7~1.8)	51(2.2,1.6~2.9) α,δ	31(1.5,1.0~2.0) δ	11(1.0,0.4~1.5)
RIF+STR+EMB	0(0.0,-)	2(1.7,-0.7~4.1)	0(0,-)	0(0,-)	17(1.0,0.5~1.4)	44(1.9,1.4~2.5) α,χ,δ	23(1.1,0.6~1.5)	7(0.6,0.2~1.1)
INH+RIF+STR+EMB	1(9.1,-8.7~26.9)	11(9.6,4.2~15)	1(1.6,-1.6~4.9)	1(10,-9.6~29.6)	137(7.8,6.5~9.0) δ	284(12.5,11.2~13.9) α,χ,δ	179(8.5,7.3~9.7) δ	49(4.3,3.1~5.5)

^1^ TB-HIV coinfected patients younger than 25 years old, *n* = 11;

^2^ TB-HIV coinfected patients between the ages of 25 and 44, *n* = 115;

^3^ TB-HIV coinfected patients between the ages of 45 and 64, *n* = 61;

^4^ TB-HIV coinfected patients older than 64 years old, *n* = 10;

^5^ TB without HIV infection patients younger than 25 years old, *n* = 1762;

^6^ TB without HIV infection patients between the ages of 25 and 44, *n* = 2267;

^7^ TB without HIV infection patients between the ages of 45 and 64, *n* = 2101;

^8^ TB without HIV infection patients older than 64 years old, *n* = 1143.

#: Resistant to at least one drug.

*: *P* < 0.05 vs. TBw/oHIV group (the same age level).

α: *P* < 0.05 vs. the < 25-year-old TBw/oHIV group;

β: *P* < 0.05 vs. the 25-44-year-old TBw/oHIV group;

χ: *P* < 0.05 vs. the 45-64-year-old TBw/oHIV group;

δ: *P* < 0.05 vs. the > 64-year-old TBw/oHIV group.

**Fig 4:**
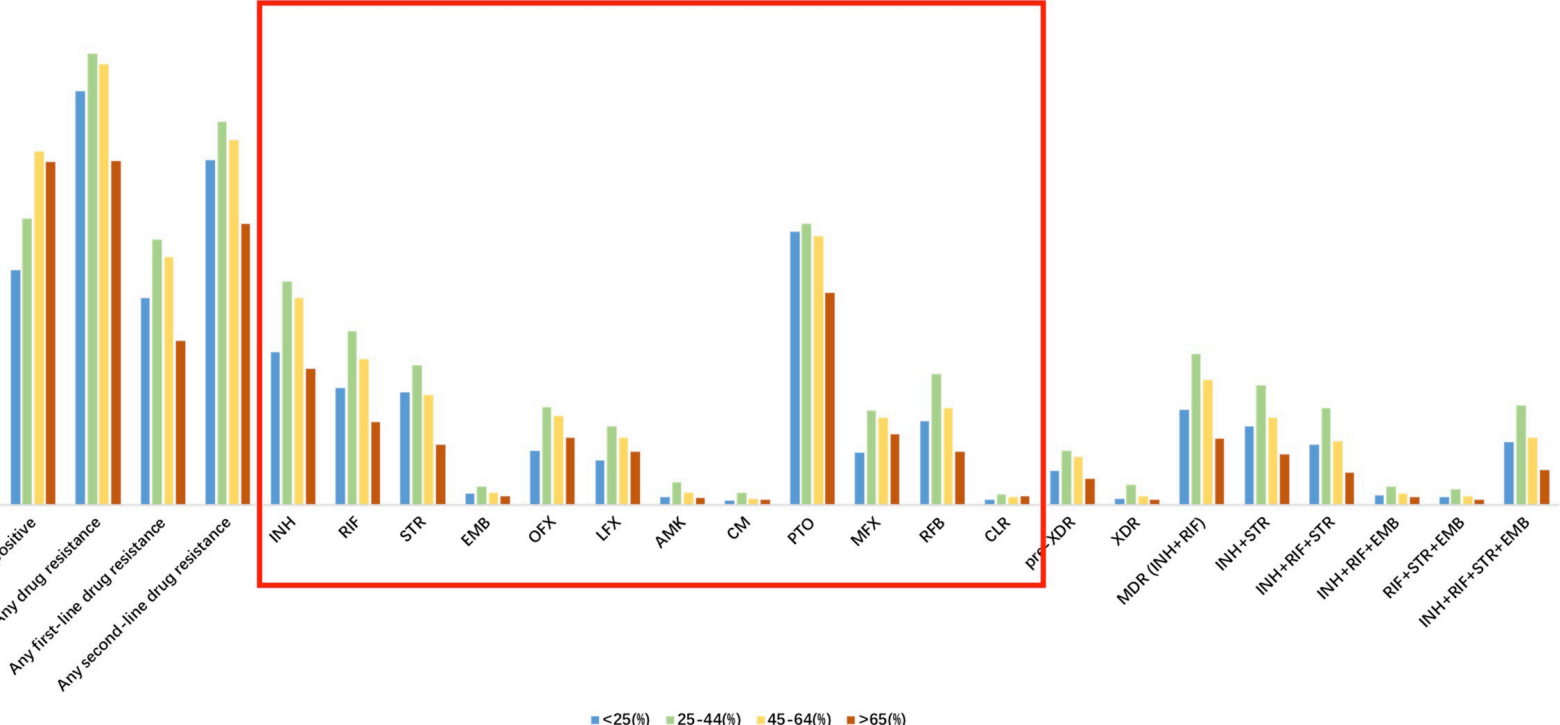
ATD profiles of all TB patients of different ages.

When the 4 first-line ATDs and 8 second-line ATDs were taken as a whole in the Wilcoxon rank-sum analysis, among TB-HIV patients, the 45~64-year-old TB-HIV patients had the highest resistance profile, which was significantly higher than that of the <25-year-old TBw/oHIV patients (*P*<0.05, **Table 2 and Fig 4**). Additionally, among TBw/oHIV patients, the 25~44-year-old TBw/oHIV patients had the highest resistance profile, which was significantly higher than those of the <25-year-old TBw/oHIV patients (*P*<0.05), the 45~64-year-old TBw/oHIV patients (*P*<0.05) and the >65-year-old TBw/oHIV patients (*P*<0.05, **Table 2 and Fig 4**). The 45- to 64-year-old TBw/oHIV patients had the second highest resistance profile, which was significantly higher those the <25-year-old TBw/oHIV patients (*P*<0.05) and the >65-year-old TBw/oHIV patients (*P*<0.05, **Table 2 and Fig 4**).

### Drug resistance profiles of different years

Patients in different years were divided into 4 groups (2014, 2015, 2016, and 2017).

**Fig 5:**
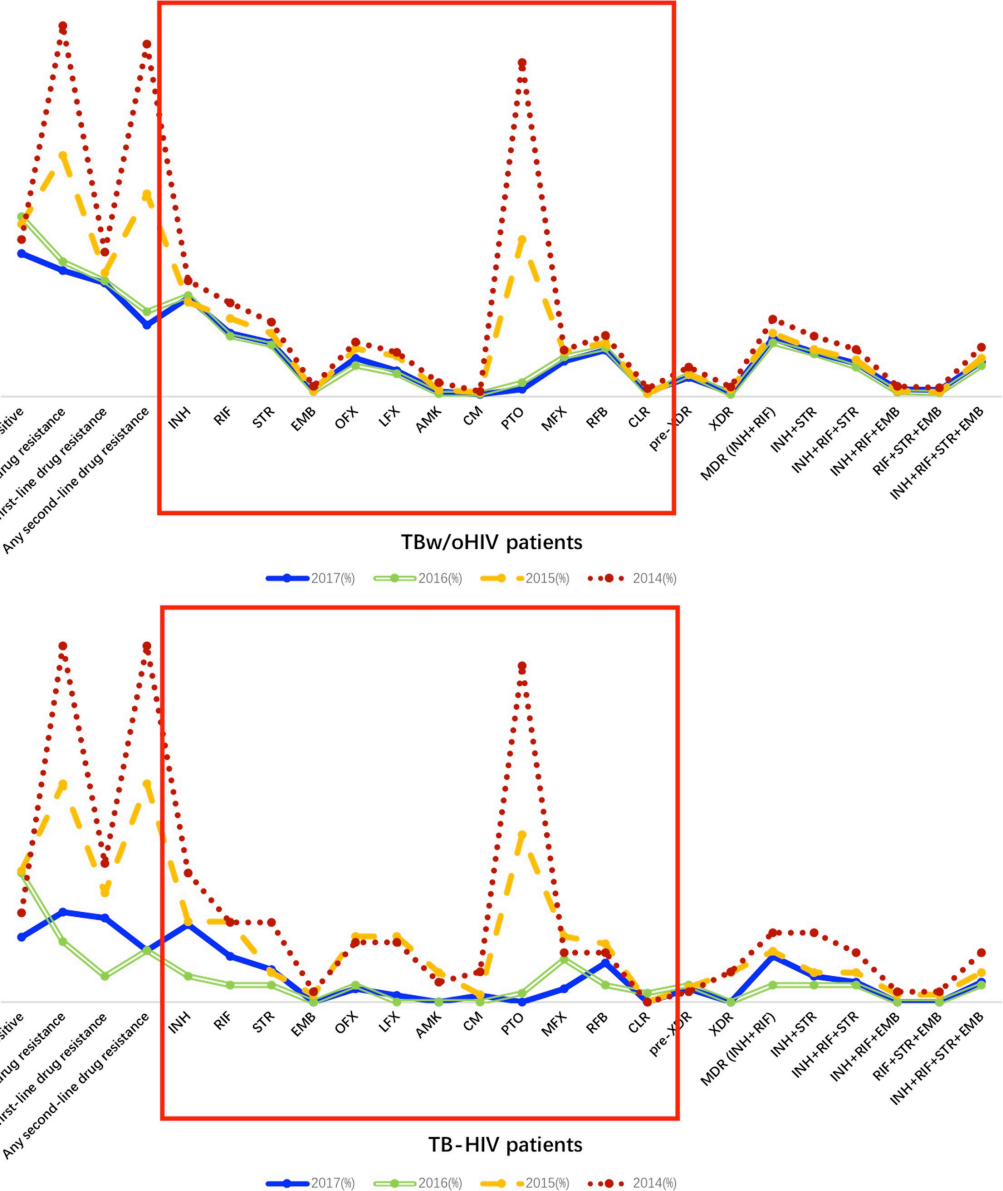
ATD profiles in different years. **5A:** ATD profiles of TB-HIV groups in different years. **5B:** ATD profiles of TBw/oHIV groups in different years.

When the 4 first-line ATDs and 8 second-line ATDs were taken as a whole in the Wilcoxon rank-sum analysis, in the TBw/oHIV group, the patients in 2014 had the highest resistance profile, which was significantly higher than those of the TBw/oHIV patients in 2015 (*P*<0.05, Table 3 and **Fig 5A**), 2016 (*P*<0.05, **Fig 5A**) and 2017 (*P*<0.05, **Fig 5A**). In addition, the resistance profile of the TBw/oHIV patients in 2015 was higher than those of the TBw/oHIV patients in 2016 (*P*<0.05, **Fig 5A**) and 2017 (*P*<0.05, **Fig 5A**).

**Table 3:** Drug-resistant distribution of Mycobacterium tuberculosis isolates in different years.

Drug resistance	TB-HIV coinfection [*N* (%, 95% CI)]				TB without HIV infection [*N* (%, 95% CI)]			
	2014^1^	2015^2^	2016 ^3^	2017^4^	2014^5^	2015 ^6^	2016 ^7^	2017^8^
AFB smear-positive	9(23.1,9.7~36.5)	18(34,21.1~46.8) **η**	15(33.3,19.4~47.3) **η**	10(16.7,7.2~26.2)*	740(36,33.9~38.1) **β,χ**	822(39.6,37.5~41.7) **δ**	809(41.3,39.1~43.4) **δ**	388(32.9,30.2~35.5)
Any drug resistance #	36(92.3,83.8~100.8)**ϕ,γ,η**	30(56.6,43.1~70.1) **γ,η**	7(15.6,4.8~26.3) *	14(23.3,12.5~34.1)	1752(85.2,83.7~86.7) **β,χ,δ**	1147(55.3,53.1~57.4) **χ,δ**	605(30.9,28.8~32.9)	342(29.0,26.4~31.5)
Any first-line drug resistance	14(35.9,20.6~51.1) **γ**	15(28.3,16.1~40.5) **γ**	3(6.7,-0.7~14) *,**η**	13(21.7,11.2~32.2)	680(33.1,31~35.1) **β,χ,δ**	591(28.5,26.5~30.4)	521(26.6,24.6~28.5)	308(26.1,23.6~28.6)
Any second-line drug resistance	36(92.3,83.8~100.8) **ϕ,γ,η**	30(56.6,43.1~70.1) **γ,η**	6(13.3,3.3~23.4)	8(13.3,4.7~22.0)	1664(80.9,79.2~82.6) **β,χ,δ**	965(46.5,44.4~48.7) **χ,δ**	382(19.5,17.7~21.2) **δ**	194(16.4,14.3~18.5)
INH	13(33.3,18.3~48.3) **γ**	11(20.8,9.7~31.8) **γ**	3(6.7,-0.7~14) *	12(20.0,9.8~30.2)	544(26.5,24.6~28.4) **β,χ,δ**	449(21.6,19.9~23.4)	453(23.1,21.2~25)	268(22.7,20.3~25.1)
RIF	8(20.5,7.7~33.4) **γ**	11(20.8,9.7~31.8) **γ**	2(4.4,-1.6~10.5)	7(11.7,3.5~19.9)	443(21.5,19.8~23.3) **β,χ,δ**	373(18,16.3~19.6) **χ,δ**	271(13.8,12.3~15.3)	171(14.5,12.5~16.5)
STR	8(20.5,7.7~33.4) **γ**	4(7.5,0.4~14.7)	2(4.4,-1.6~10.5)	5(8.3,1.3~15.4)	349(17,15.4~18.6) **β,χ,δ**	300(14.5,12.9~16) **χ**	232(11.8,10.4~13.3)	146(12.4,10.5~14.2)
EMB	1(2.6,-2.5~7.6)	1(1.9,-1.8~5.6)	0(0,-)	0(0.0,-)	50(2.4,1.8~3.1) **β,χ**	27(1.3,0.8~1.8)	23(1.2,0.7~1.6)	21(1.8,1.0~2.5)
OFX	6(15.4,3.9~26.9) **η**	9(17,6.8~27.2) **γ,η**	2(4.4,-1.6~10.5)	2(3.3,-1.2~7.9)	257(12.5,11.1~13.9) **χ,δ**	226(10.9,9.6~12.2) **χ**	140(7.1,6~8.3)	104(8.8,7.2~10.4)
LFX	6(15.4,3.9~26.9) **η**	9(17,6.8~27.2) **η**	0(0,-)	1(1.7,-1.6~4.9)	210(10.2,8.9~11.5) **χ,δ**	190(9.2,7.9~10.4) **χ,δ**	102(5.2,4.2~6.2)	68(5.8,4.4~7.1)
AMK	2(5.1,-1.9~12.1)	4(7.5,0.4~14.7) *	0(0,-)	0(0.0,-)	66(3.2,2.4~4) **β,χ,δ**	31(1.5,1~2) **χ**	12(0.6,0.3~1)	13(1.1,0.5~1.7)
CM	3(7.7,-0.8~16.2) *	1(1.9,-1.8~5.6)	0(0,-)	1(1.7,-1.6~4.9)	24(1.2,0.7~1.6)	20(1,0.5~1.4)	13(0.7,0.3~1)	6(0.5,0.1~0.9)
PTO/ETO	34(87.2,76.5~97.8) **ϕ,γ**	23(43.4,29.9~56.9) **γ**	1(2.2,-2.1~6.6)	0(0.0,-)	1579(76.8,75~78.6) **β,χ,δ**	746(36,33.9~38) **χ,δ**	63(3.2,2.4~4) **δ**	20(1.7,1.0~2.4)
MFX	5(12.8,2.2~23.5)	9(17,6.8~27.2) **η**	5(11.1,1.8~20.4)	2(3.3,-1.2~7.9)	220(10.7,9.4~12) **δ**	214(10.3,9~11.6) **δ**	179(9.1,7.9~10.4)	96(8.1,6.6~9.7)
RFB	5(12.8,2.2~23.5)	8(15.1,5.4~24.8)	2(4.4,-1.6~10.5)	6(10.0,2.3~17.7)	290(14.1,12.6~15.6) **χ,δ**	255(12.3,10.9~13.7)	219(11.2,9.8~12.6)	126(10.7,8.9~12.4)
CLR	0(0.0,-)	0(0,-)	1(2.2,-2.1~6.6)	0(0.0,-)	37(1.8,1.2~2.4) **β,χ**	12(0.6,0.3~0.9)	14(0.7,0.3~1.1)	12(1.0,0.4~1.6)
pre-XDR	1(2.6,-2.5~7.6)	2(3.8,-1.4~9)	2(4.4,-1.6~10.5)	2(3.3,-1.2~7.9)	140(6.8,5.7~7.9) **β,χ,δ**	106(5.1,4.2~6.1)	98(5,4~6)	51(4.3,3.2~5.5)
XDR	3(7.7,-0.8~16.2) *	4(7.5,0.4~14.7) *	0(0,-)	0(0.0,-)	45(2.2,1.6~2.8) **χ,δ**	33(1.6,1.1~2.1) **χ**	8(0.4,0.1~0.7)	10(0.8,0.3~1.4)
MDR (INH+RIF)	7(17.9,5.7~30.2)	7(13.2,4~22.4)	2(4.4,-1.6~10.5)	7(11.7,3.5~19.9)	364(17.7,16.1~19.4) **β,χ,δ**	302(14.6,13~16.1) **χ**	242(12.3,10.9~13.8)	157(13.3,11.4~15.2)
INH+STR	7(17.9,5.7~30.2)	4(7.5,0.4~14.7)	2(4.4,-1.6~10.5)	4(6.7,0.3~13.0)	283(13.8,12.3~15.3) **β,χ,δ**	224(10.8,9.5~12.1)	190(9.7,8.4~11)	120(10.2,8.4~11.9)
INH+RIF+STR	5(12.8,2.2~23.5)	4(7.5,0.4~14.7)	2(4.4,-1.6~10.5)	3(5.0,-0.6~10.6)	223(10.8,9.5~12.2) **β,χ,δ**	176(8.5,7.3~9.7)	133(6.8,5.7~7.9)	90(7.6,6.1~9.1)
INH+RIF+EMB	1(2.6,-2.5~7.6)	1(1.9,-1.8~5.6)	0(0,-)	0(0.0,-)	49(2.4,1.7~3) **β,χ**	27(1.3,0.8~1.8)	19(1,0.5~1.4)	20(1.7,1.0~2.4)
RIF+STR+EMB	1(2.6,-2.5~7.6)	1(1.9,-1.8~5.6)	0(0,-)	0(0.0,-)	40(1.9,1.3~2.5) **β,χ**	20(1,0.5~1.4)	15(0.8,0.4~1.2)	16(1.4,0.7~2.0)
INH+RIF+STR+EMB	5(12.8,2.2~23.5)	4(7.5,0.4~14.7)	2(4.4,-1.6~10.5)	3(5.0,-0.6~10.6)	233(11.3,10~12.7) **β,χ,δ**	183(8.8,7.6~10)	139(7.1,6~8.2)	94(8.0,6.4~9.5)

^1^*n* = 39;

^2^*n* = 53;

^3^*n* = 45;

^4^*n* = 60;

^5^*n* = 2056;

^6^*n* = 2075;

^7^*n* = 1961;

^8^*n* = 1181.

#: Resistant to at least one drug.

*: *P* < 0.05 vs. TB without HIV infection group.

**α**: *P* < 0.05 vs. 2014 TBw/oHIV group;

**β**: *P* < 0.05 vs. 2015 TBw/oHIV group;

**χ**: *P* < 0.05 vs. 2016 TBw/oHIV group;

**δ**: *P* < 0.05 vs. 2017 TBw/oHIV group.

**ε**: *P* < 0.05 vs. 2014 TB-HIV group;

**ϕ**: *P* < 0.05 vs. 2015 TB-HIV group;

**γ**: *P* < 0.05 vs. 2016 TB-HIV group;

**η**: *P* < 0.05 vs. 2017 TB-HIV group.

On the other hand, in the TB-HIV group, the patients in 2014 had the highest resistance profile, which was significantly higher than those of the TB-HIV patients in 2016 (*P*<0.05, **Fig 5B**) and the TB-HIV patients in 2017 (*P*<0.05, **Fig 5B**). Additionally, the resistance profile of the TB-HIV patients in 2015 was higher than those of the TB-HIV patients in 2016 (*P*<0.05, **Fig 5B**) and 2017 (*P*<0.05, **Fig 5B**).

As shown in Table 3, TB-HIV coinfected patients had lower resistance rates to INH than TBw/oHIV patients in 2015 (20.8% vs. 21.6%, Table 3), 2016 (6.7% vs. 23.1%, *P*<0.05, Table 3), and 2017 (20.0% vs. 22.7%, Table 3), but a statistically significant result was only observed in 2016. Although TB-HIV patients had lower INH resistance rates, they had higher XDR rates than the TBw/oHIV patients in both 2014 (7.7% vs. 2.2%, *P*<0.05) and 2015 (7.5% vs. 1.6%, *P*<0.05). For the TBw/oHIV group, the 2014 patients had higher MDR rates than the patients in 2015 (17.7% vs. 14.6%, *P*<0.05, Table 3), 2016 (17.7% vs. 12.3%, *P*<0.05, Table 3) and 2017 (17.7% vs. 13.3%, *P*<0.05, Table 3), as well as higher XDR rates than the patients in 2016 (2.2% vs. 0.4%, *P*<0.05) and 2017 (2.2% vs. 0.8%, *P*<0.05).

### Performance characteristics of the GeneXpert assay

Of the total of 7470 culture-confirmed *Mycobacterium tuberculosis* cases, 815 cases received a GeneXpert assay, and 3 of these GeneXpert assays failed. The GeneXpert TB-positive rate in the TBw/oHIV group was higher than that in the TB-HIV group [81%(639/792) vs. 65% (13/20), *P* = 0.08]. As shown in Table 4, of all 812 cases with DST results and GeneXpert assay data, 148 cases were identified as involving RIF-resistant TB according to the conventional culture method. In comparison, with the GeneXpert assay, a total of 120 cases were found to involve RIF-resistant TB. The sensitivity, specificity, positive predictive value (PPV) and negative predictive value (NPV) of Xpert MTB/RIF in the detection of RIF resistance in comparison to those of the conventional phenotypic drug susceptibility technique were found to be 81.1%, 94.6%, 76.9% and 95.7%, respectively (Table 4). Similar results were also found in the TBw/oHIV group. Among all cases, the agreement in results between Gene Xpert MTB/RIF and the M960 system was 92.1% and the Kappa value was 0.74. Notably, in TBw/oHIV cases, the agreement was 92.3% and the Kappa value was 0.75. In TB-HIV cases, the agreement was 85.0% and the Kappa value was 0.32.

**Table 4:** Performance characteristics of the GeneXpert assay compared to drug susceptibility testing for rifampicin (RIF).

	TBw/oHIV & TB-HIV					TBw/oHIV					TB-HIV				
Xpert MTB/RIF	DST-RIF resistant	DST-RIF sensitive	Total	PPV(%)	NPV(%)	DST-RIF resistant	DST-RIF sensitive	Total	PPV(%)	NPV(%)	DST-RIF resistant	DST-RIF sensitive	Total	PPV(%)	NPV(%)
GX-RIF(+)	120	36	156	76.9		119	35	154	77.3		1	1	2	50	
GX-RIF(-)	28	628	656		95.7	26	612	638		95.9	2	16	18		88.9
Total	148	664	812			145	647	792			3	17	20		
Sensitivity(%)	81.1					82.1					33.3				
Specificity(%)		94.6					94.6					94.1			

DST, drug susceptibility testing; MTB, *Mycobacterium tuberculosis*; RIF, rifampicin; GX, GeneXpert; NPV, negative predictive value; PPV, positive predictive value.

## Discussion

To our knowledge, this is the first study to examine DR-TB trends over a recent period of time (January 2014 to December 2017) in Sichuan, China. And there is insufficient information about the performance of the GeneXpert assay among TBw/oHIV patients and TB-HIV patients in southwest China. The data were collected in PHCCC, which is also called Chengdu Tuberculosis Control Hospital. This is the leading Tuberculosis Hospitals Union with the most accurate measurements and standardized treatment in southwest China.

### General DR-TB in Sichuan

Tuberculosis, a refractory disease, has always been the focus of disease control and prevention in China. In our study, we analyzed the data from four-year consecutive TB patients and found that the TB drug resistance status was more serious than imagined in Sichuan and that the drug resistance rate was higher than that at the general level in China; this was especially true for the MDR resistance rate, which was approximately 2 times higher than that at the national level (14.6% vs. 6.8%) [2]. Our data suggest that the prevalence of MDR-TB in Sichuan is serious. The high burden of MDR-TB cases in Sichuan might be due to the mountainous and remote geographic characteristics of the region, the underdeveloped economy, the large migrant population, the relatively low average educational level, the unstable quality of DOTS, an inability to obtain timely and effective treatment, and poor infection control [11–13].

Concerning treatment history, in our study, the proportion of MDR-TB among all cases was 14.6%, which was also higher than the national level (10%) [14] and the global level (6.2%) [1]. In recent years, several studies have investigated local TB resistance rates throughout the country. The results indicated that the MDR rate ranged from 7.68% to 24.13%, among which the resistance rate of the first-time-admitted cases was 3.68 ~19.49%. The resistance rate of repeatedly admitted cases was 22.50 ~ 42.45%. More results based on large multicenter investigations are still needed.

### MDR-TB/XDR-TB status

In this study, the proportion of MDR-TB cases with XDR-TB was 9.0% for the TBw/oHIV group, 30.4% for the TB-HIV group, and 9.5% (103/1088) for all patients. A recent DR-TB survey in Sichuan from 2011 to 2015 demonstrated that XDR-TB patients accounted for 18.1% of MDR-TB patients [6]. The difference might be mainly explained by the different study periods (2011–2015 vs. January 2014-December 2017), study subjects (6158 smear-positive patients *vs*. 7470 isolated cases with only 2811 smear-positive cases), the sampling regions (12 cities and 108 counties in Sichuan *vs*. the PHCCC), and other factors. In contrast, the XDR-TB in our results (9.5%) was lower than that in the results from Tang, in which XDR-TB cases accounted for 28.8% of MDR-TB cases. In this report, data were collected from different areas of China [15]. Recently, a retrospective study showed that XDR-TB accounted for 27.9% of MDR-TB in Shandong Province, which is located in eastern China [16]. Another study from Hunan Province, which is located in the center of China, showed that XDR accounted for only 2.1% of MDR-TB. Geographical variation might be considered an impact factor for the ratio of XDR-TB/MDR-TB. Our study revealed that in Sichuan province, the ratio of XDR-TB in the TB-HIV group was significantly higher than those in both the TBw/oHIV group (30.4% vs. 9.0%, *P*<0.05) and all TB patients (9.0% vs. 10.1%, *P*<0.05). XDR-TB might still be underestimated in southwest China. Thus, this study might be helpful as an alert for the inefficient TB control and management of XDR-TB in southwest China.

### The DR-TB status in different ages

With respect to age, the <25-year-old TB-HIV patients had a significantly higher XDR ratio than the <25-year-old TBw/oHIV patients (9.1% vs. 0.7%, P<0.05). The 25- to 44-year-old TB-HIV patients had a significantly higher XDR proportion than the 25- to 44-year-old TBw/oHIV patients (5.2% vs. 2.4%, *P*<0.05). These results indicate that TB-HIV patients, especially the <45-year-old group, might be at high risk of XDR-TB. Thus, it is necessary to pay more attention to TB-HIV patients and strengthen the management of their treatment process to improve their cure rate.

Regarding the different age groups of TBw/oHIV patients, the 25~44-year-old TBw/oHIV patients had the highest MDR and XDR proportions and the highest resistance profile. The 45- to 64-year-old TBw/oHIV patients had the second highest resistance profile. Unsurprisingly, the drug resistance cases were more likely to be found in the 25~44-year-old group and 45~6-year-old 4 group. Sichuan is one of the largest labor output provinces in China. The total labor output increased from 24.14 million in 2012 to 24.91 million in 2016 [17]. People aged 25~44 years and 45~64 years are the major labor force. These people are characterized by strong mobility, more exposure factors, and less compliance, which subsequently results in incomplete and nonstandard treatment. For this reason, they may become an important source of infection by drug-resistant tuberculosis.

### The DR-TB status for different years

Regarding different years, the ATD-resistance profile of the TBw/oHIV patients presented as 2014>2015>2016≈2017 (**Fig 5A**). In parallel, the same trend was observed for the TB-HIV patients: 2014>2015>2016≈2017 (**Fig 5B**). These data indicate that the control of drug resistance is strengthened, and the effort to combat tuberculosis is effective in Sichuan. However, we are a long way from winning this battle.

### The DR-TB status in HIV patients

Our valuable clinical data contains 197 recruited TB-HIV patients. This will help us understand and prevent tuberculosis drug resistance among TB patients who are coinfected with HIV. TB-HIV coinfection has always been associated with high rates of TB drug resistance [18]. To our surprise, all TB-HIV patients had a lower drug resistance rate than all TBw/oHIV patients (44.2% vs. 52.9%, *P*<0.05, Table 1). Moreover, the Wilcoxon rank-sum test showed that the general drug resistance rates in the repeatedly admitted TB-HIV patients were less serious than the repeatedly admitted TBw/oHIV patients (*P*<0.05, Table 1), and there was no significant difference between the first-time-admitted TB-HIV patients and the first-time-admitted TBw/oHIV patients. This finding indicated that the TB-HIV patients, especially the repeatedly admitted group, might receive better medication than the TBw/oHIV group. In addition, the TB-HIV patients showed lower resistance rates to INH than the regular TB patients. Whether some TB-HIV patients refused to receive INH because of its side-effect [19] or whether antiviral drugs can increase the efficacy of INH remains unknown. Our results suggest that INH can be more effective as a routine tuberculosis medicine for TB-HIV patients than for TBw/oHIV patients in Sichuan. However, HIV patients account for only a small percentage of the total sample size, and more research with a large population is needed to obtain more accurate data.

### Variations in the AFB smear positivity rate among different groups

First, we found that the positivity rate of the AFB smear in TB-HIV patients was significantly lower than that in the TBw/oHIV patients (*P*<0.05, **Fig 2**). Our result is similar to that of a previous report. Palmieri found that compared to HIV-negative patients, TB patients with HIV more often present with negative AFB sputum smears [20]. Thus, when the AFB results are negative, more detections are needed to exclude the possibility of TB infection, especially in patients with a high risk of HIV.

### Evaluation of GeneXpert for detecting RIF resistance of respiratory Mycobacterium tuberculosis

In Sichuan, GeneXpert assays were performed at patients' own expense, with a cost of approximately 110USD for one person. This expense might be the main reason why of the total of 7470 culture-confirmed TB cases, only 11% received the GeneXpert assay.

The GeneXpert assay has high sensitivity and specificity for the diagnosis of TB, especially for TBw/oHIV patients [21], which was confirmed in our study. However, we found that for TB-HIV patients, the GeneXpert TB-positive rate was significantly lower than that in TBw/oHIV patients [65% (13/20) vs. 81% (639/792), *P*<0.05]. Thus, Xpert MTB/RIF Ultra, whose sensitivity was superior to that of the GeneXpert assay, was recommend for TBw/oHIV patients [22].

In our study, the agreement between the GeneXpert assay and the M960 system with respect to RIF resistance was substantial in TBw/oHIV cases (92.3%; Kappa value, 0.75) but only fair in TB-HIV cases (85.0%; Kappa value, 0.32), indicating that the GeneXpert assay is a simple, rapid, and accurate test for detecting RIF resistance in TBw/oHIV patients, whereas the accuracy of GeneXpert is inadequate in patients with HIV for the detection of RIF resistance.

In our study, 28 cases were negative with GeneXpert assay but positive with the M960 system (Table 4). The reason why RIF resistance of some TB cases was detected by the M960 system but not by the GeneXpert assay may be that five rpoB molecular beacons used in GeneXpert could detect mutations responsible for approximately 95% of RIF-R [23], while some TB cases might involve new mutants that could not be detected by the GeneXpert assay. Additionally, the GeneXpert assay requires approximately 130 bacilli/mL of samples, whereas M960 culture requires as little as 10 to 100 bacilli/mL [24]. Thus, the concentration of TB in certain samples might be under the detection limit of the GeneXpert assay. However, 36 cases were positive according to the GeneXpert assay but negative according to the M960 system (Table 4). We assumed that the DNA of live and dead TB could both be detected by the GeneXpert assay, while only living TB could be detected by the M960 system. Some living TB might have been extinguished by sodium hydroxide in the pretreatment step and thus could not be identified by the M960 culture method.

### Limitation

Limitations of our findings were as follows. First, because the study was based on a retrospective study design, we did not have contact with the patients, some important variables which related to the underlying causes of drug resistance, including detailed TB treatment histories; the type of medications; the side effects of the drugs; blood test results, such as hemoglobin and liver function; and detailed molecular typing data were not available in the registers. Second, this study was carried out in 1 provincial hospital in Sichuan. We did not include data from CDC clinics, other hospitals or other health facilities. Third, the DST panel does not include all ATDs. This might have led to an inaccurate estimation of the overall TB status in Sichuan province.

## Conclusion

This study summarizes the most recent DR-TB status against first- and second-line drugs in TB patients with and without HIV coinfections in Sichuan province, China. Although the status of ATD-resistance has improved since 2014 (Table 3 and **Fig 4A** and **4B**), it remains severe, especially for the prevention of MDR-TB in TBw/oHIV patients and XDR-TB in TB-HIV patients (Table 2). ATDs constitute future key strategies for controlling TB in Sichuan. Our findings indicate that TB-HIV patients and TBw/oHIV patients might have different ATD-resistance profiles and need different treatments. GeneXpert assay might be more suitable for TBw/oHIV patients than for TB-HIV patients. Further detailed genomic analysis is required to evaluate transmission patterns and mutations that may affect resistance to ATDs.

## Supporting information

S1 Table Drug sensitivity testing and GeneXpert assay data. TBwoHIV: Drug sensitivity testing and GeneXpert assay data in the TB without HIV infection group; TB-HIV: Drug sensitivity testing and GeneXpert assay data in the TB-HIV coinfection group.
(XLSX)
